# Traumatic brain injury augurs ill for prolonged deficits in the brain’s structural and functional integrity following controlled cortical impact injury

**DOI:** 10.1038/s41598-021-00660-5

**Published:** 2021-11-03

**Authors:** Abdalla Z. Mohamed, Paul Cumming, Fatima A. Nasrallah

**Affiliations:** 1 The Queensland Brain Institute, The University of Queensland, Building 79, Upland Road, Saint Lucia, Brisbane, QLD 4072 Australia; 2 Thompson Institute, University of the Sunshine Coast, Birtinya, QLD 4575 Australia; 3 Department of Nuclear Medicine, Bern University Hospital, Bern, Switzerland; 4 School of Psychology and Counselling, Queensland University of Technology, Brisbane, Australia

**Keywords:** Neuroscience, Diseases of the nervous system

## Abstract

Previous neuroimaging studies in rodents investigated effects of the controlled cortical impact (CCI) model of traumatic brain injury (TBI) within one-month post-TBI. This study extends this temporal window to monitor the structural–functional alterations from two hours to six months post-injury. Thirty-seven male Sprague–Dawley rats were randomly assigned to TBI and sham groups, which were scanned at two hours, 1, 3, 7, 14, 30, 60 days, and six months following CCI or sham surgery. Structural MRI, diffusion tensor imaging, and resting-state functional magnetic resonance imaging were acquired to assess the dynamic structural, microstructural, and functional connectivity alterations post-TBI. There was a progressive increase in lesion size associated with brain volume loss post-TBI. Furthermore, we observed reduced fractional anisotropy within 24 h and persisted to six months post-TBI, associated with acutely reduced axial diffusivity, and chronic increases in radial diffusivity post-TBI. Moreover, a time-dependent pattern of altered functional connectivity evolved over the six months’ follow-up post-TBI. This study extends the current understanding of the CCI model by confirming the long-term persistence of the altered microstructure and functional connectivity, which may hold a strong translational potential for understanding the long-term sequelae of TBI in humans.

## Introduction

A traumatic brain injury (TBI) event initiates a series of deleterious consequences, including neuronal cell loss, axonal damage, neuroinflammation, and demyelination^1,2^. The controlled cortical impact (CCI) model is a rodent TBI model entailing the application of a direct mechanical deformation to the brain. The CCI and other animal models of TBI capture many of the ongoing pathologies that are typically seen in human TBI survivors^3,4^. An extensive number of studies have focused on characterising the pathological cascades along with physical and behavioural impairments (i.e., motor function, spatial memory, and novel-object recognition) arising in the CCI model, extending from the initial hours to as much as a year after the injury^5^. Some immunohistochemistry investigations reported axonal injury and axonal swelling within hours to days following TBI^6,7^, along with a neuroinflammatory response as reflected by increased microglia and astrocyte activation within days post-TBI^8^ that persisted up to one year post-injury^9,10^. Other immunohistochemistry investigations showed demyelination processes to initiate within a week post-TBI and persist to 28 days^2^, two months^11^, six months^12^, and one year^13^.

In the CCI model, studies reported structural deformations with a lesion visible as a T2-weighted hyperintense region within hours post-TBI and expanded with time to reach the ventricular margins^8,14,15^. A progressive loss of neocortical and hippocampal volume in the rodent brain extending from one week to one year post-CCI has also been noted^10,16^. The hippocampus is particularly vulnerable to TBI-induced damage in humans^17–20^ and animal models of TBI^21,22^. ‬‬‬‬Observations of such changes are necessary at a macroscale since microscale changes are hard to detect using conventional structural MRI.

Diffusion Tensor Imaging (DTI) has become an invaluable tool for the assessment of microstructural alterations in clinical TBI studies^23,24^ and in experimental animal models^2,25,26^. DTI revealed reduced white matter integrity as reflected by lower fractional anisotropy (FA) and increased diffusivity measures in major white matter tracts in rodent brain up to one month post-TBI^2,8^. These DTI alterations correlated with the extent of axonal injury within hours post-TBI^6,25^, and with the degree of neuroinflammation^8,11^ and the extent of demyelination up to one month post-TBI^2,25^. In addition, the resting state functional MRI (rsfMRI) technique provides an index of the extent of perturbed connectivity in the rodent CCI model^27–30^ and the fluid-percussion injury models^31,32^. Those studies consistently showed decreased functional connectivity ipsilateral to the injury and increased functional connectivity in contralateral brain structures in the motor circuits, sensory cortex, cerebellum, basal ganglia, and thalamus up to one month following TBI^4,27–30^.

To date, most rodent MRI studies of brain structure and function have highlighted early phase alterations following a TBI. Very roughly, one month in the life of a rat might correspond to two years of the human lifespan^33^, whereas a human TBI can cause disability lasting for decades. Insofar as rodent studies suggest a prolonged phase of functional reorganization of the injured brain, we see a need for follow-up studies with appropriate temporal scaling relative to the human lifespan, in order to enhance the translational significance of the preclinical research. With this in mind, we tested the hypothesis that longitudinal fMRI up to six months after CCI would reveal detailed temporo-spatial mapping of early, middle, and late phases of the reorganization of rate brain after a TBI event.

## Results

The CCI animals experienced brief seizures (< 15 s) following the TBI, which manifested either in tail twitching or generalised seizures upon initial recovery from the impact. Compared to the sham animals, the TBI animals took longer to regain consciousness following the surgery (6.0 ± 1.5 min vs. 3.2 ± 2.4 min, p = 0.04). Compared to the sham animals, the TBI animals suffered a slight weight loss at day1 post-TBI (14.5 ± 6.14 gm vs. 8.7 ± 4.50 gm, p = 0.05), but this difference was not significant at day3 (11.9 ± 6.97 gm vs. 7.8 ± 8.30 gm, p = 0.3). Moreover, compared to the sham group, the TBI group showed signs of weakness at the two hours, day1, and day3 scanning sessions. From day7 onwards, there were no overt behavioural changes in any of the CCI or sham animals, all of which showed normal grooming, weight gain, motor movements, and alertness.

### Structural data showed increased lesion area and reduced volumes of different brain structures in CCI rats

**Figure 1:**
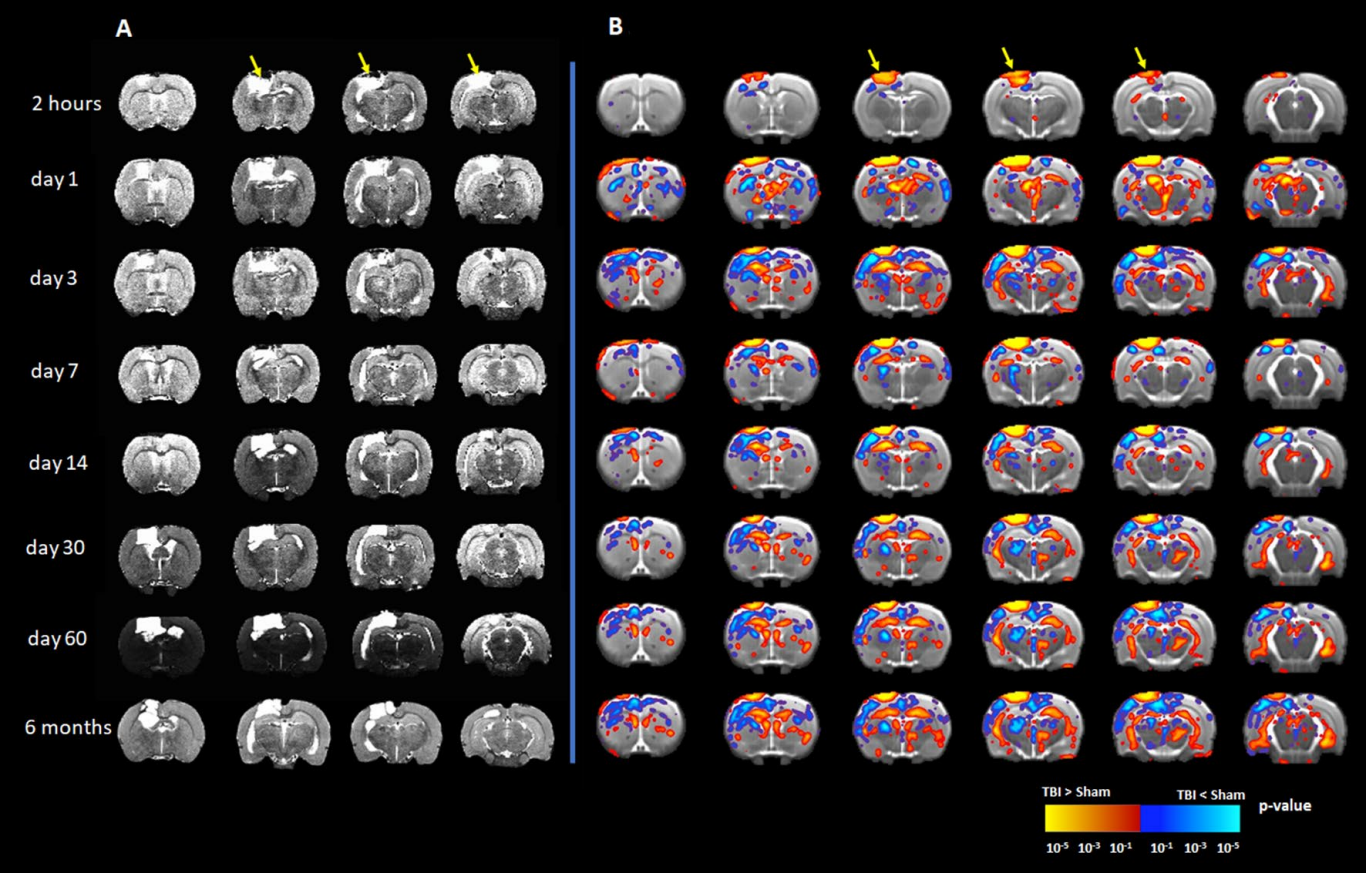
The T2 MRI data shows alterations of brain volume following controlled cortical impact (CCI) injury that persisted for six months post-TBI. (**A**) A representative set of T2 images from a single animal centred around the lesion site shows the progression of the lesion over time, with associated ventricular dilation and increases in oedema volume. (**B**) The group differences between the TBI and sham using tensor-based morphometry (family wise error corrected; p ≤ 0.05) revealed the brain atrophy due to CCI over the six months period. The images showed ventricular dilation and brain atrophy of the ipsilateral cortex, hippocampus, thalamus and caudate-putamen, which extended over time to involve the contralateral hemisphere. The yellow arrows point to the lesion area. Red-yellow represents increased volume in the TBI group, while blue-green represents reduced volume in the TBI groups as compared to sham group.

The T2-weighted images of CCI rats showed increasing lesion volumes with time, indicative of progressive oedema at the injury site, as represented by the high-intensity pixels at the grey matter and white matter interface, with no such changes in tissue contrast in the sham animals (Fig. 1). As shown in a representative animal in Fig. 1A, the temporal profile of the injury progression shows an oedema-typical signal hyperintensity in and around the injury site, first appearing within 24 h post-TBI, and partially resolving at day3. Starting at day7, the centre of the lesion began to show signal hyperintensity, which extended over time towards the ventricle margin at day14 onwards, with a distinct boundary between the lesion cavity and the surrounding tissue developing over time.

The voxel-based tensor-based morphometry revealed ventricular dilation with onset at day1 post-TBI and persisting at six months in the TBI group (p ≤ 0.05; Fig. 1B). Meanwhile, there was a significant reduction of the cortical volume mainly surrounding the lesion area at 2 h post-TBI (p ≤ 0.05), which expanded bilaterally from day1 post-TBI onwards (p < 0.01) and persisted to six months (p < 0.01; see Fig. 1B). The anterior portion of the ipsilateral hippocampus, located under the CCI target, showed damage when the lesion had expanded from the impact site to reach the lateral ventricle (p ≤ 0.05). The ipsilateral thalamus and caudate putamen both showed volume loss as of day1 (p ≤ 0.05). Volume loss propagated to the contralateral hemisphere at day3 (p ≤ 0.05) and persisted at six months (p < 0.01).

### Diffusion data revealed persistent reduced FA values in the white matter

**Figure 2:**
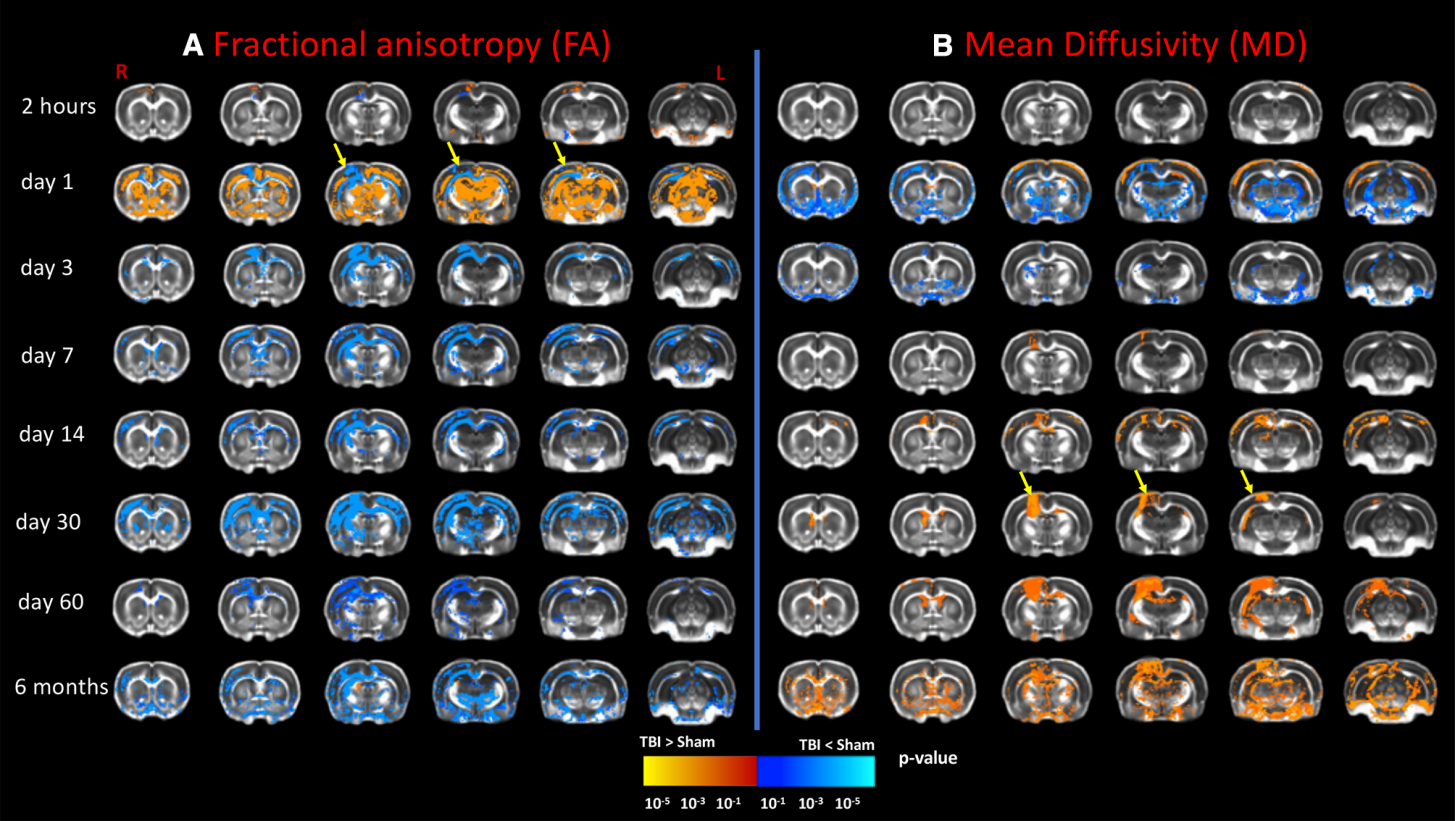
The voxel-wise analysis results from the statistical differences (two-sample t-test) of the DTI measures between the TBI and sham groups as a function of time after injury to the right hemisphere. Results showed altered white matter and grey matter microstructure following TBI in (**A**) fractional anisotropy (FA) and (**B**) mean diffusivity (MD) images. (**A**) As compared to sham group, the longitudinal DTI analysis revealed reduced fractional anisotropy (FA) in the TBI group at different white matter tracts at from day1 and up to six months post-TBI, along with increased FA values in grey matter at two hours and day1 followed by widespread FA reduction in the grey matter up to six months post-TBI. In addition, longitudinal DTI reveals acute reduction in MD during the first three days post-TBI, followed by MD increase in white matter tracts beginning at day7 and persisting up the six months post-TBI. Moreover, MD values decline in grey matter at days 1 and 3, and then increased persistently until six months post-TBI. The yellow arrows point to the lesion area. All results were corrected for multiple comparisons using family wise error correction with p ≤ 0.05. Red-yellow represents increased DTI measures (FA and MD) in the TBI group as compared to sham group, while blue-green represents reduced DTI measures (FA and MD) in the TBI groups as compared to sham group.

Figure 2 represents the temporal changes in fractional anisotropy (FA; Fig. 2A) and mean diffusivity (MD; Fig. 2B) in the rat brain. Compared to the sham group, TBI rats showed reduced FA in the ipsilateral cingulum and extending to the corpus callosum, from Bregma + 1 to − 3 mm in the AP axis at two hours post-injury. At day1, there was a further reduction in FA in the corpus callosum, cingulum, and external capsule (EC), at regions extending from Bregma + 2 mm AP to the posterior, midline retrosplenial cortex around Bregma − 4.3 mm AP (p ≤ 0.05, Fig. 2A). These regions also showed reduced FA at day3, when there was also reduced FA values in the hippocampal commissure, optic tract, IC, and anterior commissure (AC). At day7, FA was reduced in the bilateral corpus callosum, cingulum, hippocampal commissure, optic tract, EC, internal capsule (IC), and AC (p < 0.01), which persisted at day14 (p ≤ 0.05) and day30 (p < 0.01). At day60, the TBI group showed reduced FA in the bilateral corpus callosum, cingulum, hippocampal commissure, optic tract, EC, IC, and AC (p < 0.01, Fig. 2A).

In the grey matter, we saw increased FA in the lesion area at two hours post-TBI, followed by a reduction in FA compared to shams at day1 that persisted to six months post-TBI. These reductions extended from Bregma + 1 to − 3 mm AP. Increased FA at day1 was also present in the cortex, caudate-putamen, thalamus, hypothalamus, amygdala, and hippocampus, extending from Bregma + 3 mm to − 4.3 mm AP (p < 0.01). An overall reduction in FA was seen from day3 in the TBI group, including the ipsilateral cortex (p ≤ 0.05) and encompassing bilateral cortex and hippocampus at day7 (p ≤ 0.05), and bilateral cortex, hippocampus, and thalamus at day14 (p ≤ 0.05). FA was reduced in the cortex, caudate-putamen, thalamus, hippocampus, amygdala, hypothalamus, and midbrain of the TBI group at day30, in the cortex, caudate-putamen, thalamus, and amygdala at day60 (p ≤ 0.05), and in cortex, caudate putamen, thalamus, hippocampus, amygdala, hypothalamus, and midbrain at six months (p < 0.01).

### Diffusion data revealed alterations of the diffusivity measures both in white matter and grey matter

Measures of diffusivity (i.e., AD, RD, and MD) were also altered post-TBI. In the white matter, there were no significant differences in MD between the groups at two hours post-TBI, while at day1 the MD was increased in the corpus callosum and reduced in the AC, hippocampal commissure, cingulum, optic tract, and IC, and EC in the TBI group (p ≤ 0.05, Fig. 2B). Reduced MD was seen in the corpus callosum, AC, cingulum, optic tract, and IC at day3 post-TBI, but on day7 there was an increase in MD in the ipsilateral corpus callosum below the injury lesion (p ≤ 0.05) that persisted and expanded to also involve the cingulum, optic tract, and EC at day14 (p ≤ 0.05). A further increase was observed in the corpus callosum, cingulum, IC, and EC at day30, which extended bilaterally at day60 post-TBI (p ≤ 0.05). At six months, there were more extensive MD increases in the bilateral corpus callosum, cingulum, hippocampal commissure, optic tract, IC, EC, and AC (p < 0.01, Fig. 2B).

MD in the cortical lesion showed no difference compared to the sham group at two hours, was reduced at day1, showed no difference at day3, but increases at day7 persisted up to six months post-TBI (p ≤ 0.05, Fig. 2B). As for the rest of the grey matter, compared to the sham group, the TBI group showed no MD differences at two hours post injury, but had reduced MD in cortex, thalamus, hippocampus, hypothalamus, and amygdala at day1 (p < 0.01, Fig. 2B), in the amygdala, thalamus, and hippocampus at day3 (p ≤ 0.05), but no differences at day7 (p > 0.05). There was relatively increased MD in the cortex at day14 (p ≤ 0.05), which persisted at day30 and expanded at day60 to cover the cortex and hippocampus (p < 0.01). At six months, the TBI group showed a wide-spread increase in MD in the bilateral cortex, caudate-putamen, thalamus, hippocampus, amygdala, hypothalamus, and midbrain (p < 0.01).

**Figure 3:**
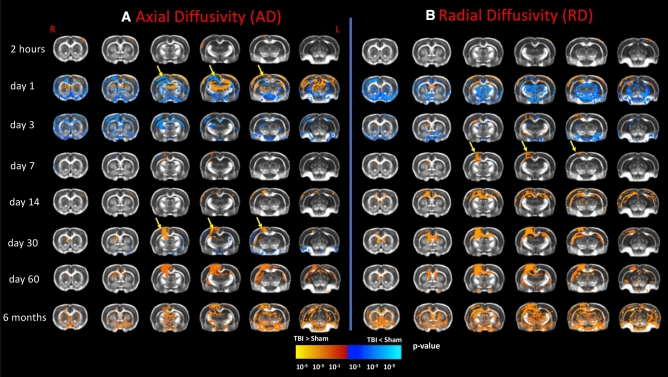
The voxel-wise analysis showing the statistical differences (two-sample t-test) for the comparison between TBI and sham groups in the diffusivity measures, which reveals altered white matter and grey matter microstructure following right sided TBI as revealed by changes in (**A**) axial diffusivity (AD) and (**B**) radial diffusivity (RD). The yellow arrows point to the lesion area. Longitudinal DTI revealed no group differences in AD at two hours, reduced AD in white matter tracts at days 1 and 3, followed by a temporary recovery at days 7 and 14, and then increased AD values at day30 that persisted up to six months post-TBI. The grey matter showed a slightly different temporal pattern with acutely increased AD at day1, decreased AD at day3, normalization at days 7, 14 and 30, and persistently increased AD from day60 up to six months. The RD followed a similar trend of the AD, with more pronounced changes at late time-points. All results were corrected for multiple comparisons using family wise error correction with p ≤ 0.05. Red-yellow represents increased diffusivity measures (i.e., AD and RD) in the TBI groups as compared to sham group, while blue-green represents reduced diffusivity measures (i.e., AD and RD) in the TBI groups as compared to sham group.

Figure 3 depicts the corresponding changes in the AD and RD diffusion metrics. In the white matter, there was no difference in AD at two hours in the TBI group compared to shams, but there was reduced AD in the TBI group at day1 in bilateral corpus callosum, cingulum, hippocampal commissure, optic tract, IC, EC, and AC (p ≤ 0.05, Fig. 3A). At day3, there was reduced AD in the bilateral corpus callosum, cingulum, hippocampal commissure, optic tract, and EC in regions underlying the motor and sensory cortical regions from Bregma + 2 to − 4.3 mm AP (p ≤ 0.05, Fig. 3A). At day7, no significant group differences were observed except for a decrease/increase in the corpus callosum just below the injury lesion, which persisted to day14 (p > 0.05). There was increased AD at day30 in the ipsilateral corpus callosum, IC, and EC, but reduced AD in the contralateral IC and EC (p ≤ 0.05). At day60, the AD was increased in the ipsilateral corpus callosum, cingulum, hippocampal commissure, and EC in the TBI group (p < 0.01), and at six months there was increased AD in most of the major white matter tracts, including the bilateral corpus callosum, cingulum, hippocampal commissure, optic tract, IC, EC, and AC in the TBI group (p < 0.01).

The cortical lesion area showed no difference in AD at two hours after TBI, but had reduced AD at days 1 and 3, was unchanged at day7, followed by increase at day14 that persisted up to six months post-TBI at the lesion area (p < 0.01, Fig. 3A). Among other grey matter regions, the TBI group showed no significant differences at two hours, but reduced AD in the cortex and amygdala in association with increased AD in the thalamus, caudate-putamen, thalamus, hypothalamus, piriform cortex, and hippocampus at day1 (p < 0.01). The reduced AD in the bilateral cortex, piriform cortex, and amygdala persisted at day3 (p ≤ 0.05), but only a small cluster of reduced AD voxels was evident in the ipsilateral cortex close to the injury at day7, and no differences in grey matter AD at days 14 and 30 post-injury (p ≤ 0.05). However, there was increased AD in the bilateral hippocampus at day60 (p ≤ 0.05), which was more widespread at six months, encompassing the bilateral caudate putamen, thalamus, hippocampus, amygdala, hypothalamus, and midbrain (p < 0.01).

In the white matter, there were no significant difference in RD at two hours post-TBI. At day1, RD was increased in the bilateral corpus callosum and EC, and reduced in the cingulum, hippocampal commissure, optic tract, and IC in the TBI group (p ≤ 0.05, Fig. 3B). On day3, the TBI group showed increased RD in the ipsilateral corpus callosum, cingulum, IC, and EC, along with reduced RD in the contralateral cingulum (p ≤ 0.05). Compared to the sham group at day7, the TBI group showed increased RD in the ipsilateral corpus callosum just below the lesion (p ≤ 0.05), that had expanded at day14 to show increased RD in the bilateral corpus callosum, cingulum, hippocampal commissure, and EC (p ≤ 0.05). The TBI group showed a persistent RD increase in the bilateral corpus callosum, IC, EC, and hippocampal commissure at days and 60 (p < 0.01), with widespread increases at six months in the bilateral corpus callosum, cingulum, hippocampal commissure, optic tract, IC, EC, and AC (p < 0.01).

In the lesion area, there were no significant changes in RD at two hours post-TBI, but there was focally reduced RD at day1, and increased RD at day3 that extended spatially with time and persisted at six months post-TBI (p ≤ 0.05, Fig. 3B). In other grey matter regions, no changes in RD were seen at two hours in the TBI group, but there was reduced RD in the cortex, caudate putamen, thalamus, hippocampus, hypothalamus, and amygdala at day1 (p < 0.01), and a less pronounced reduction in the same regions at day3 (p ≤ 0.05), which normalised at day7 (p > 0.05). There was increased RD at day14 in the cortex, and hippocampus (p ≤ 0.05), which persisted up to day60 (p ≤ 0.05), and expanded at six months post-TBI to include the bilateral cortex, caudate putamen, thalamus, hippocampus, amygdala, hypothalamus, and midbrain (p < 0.01).

### TBI caused alterations in global measures of connectivity

**Figure 4:**
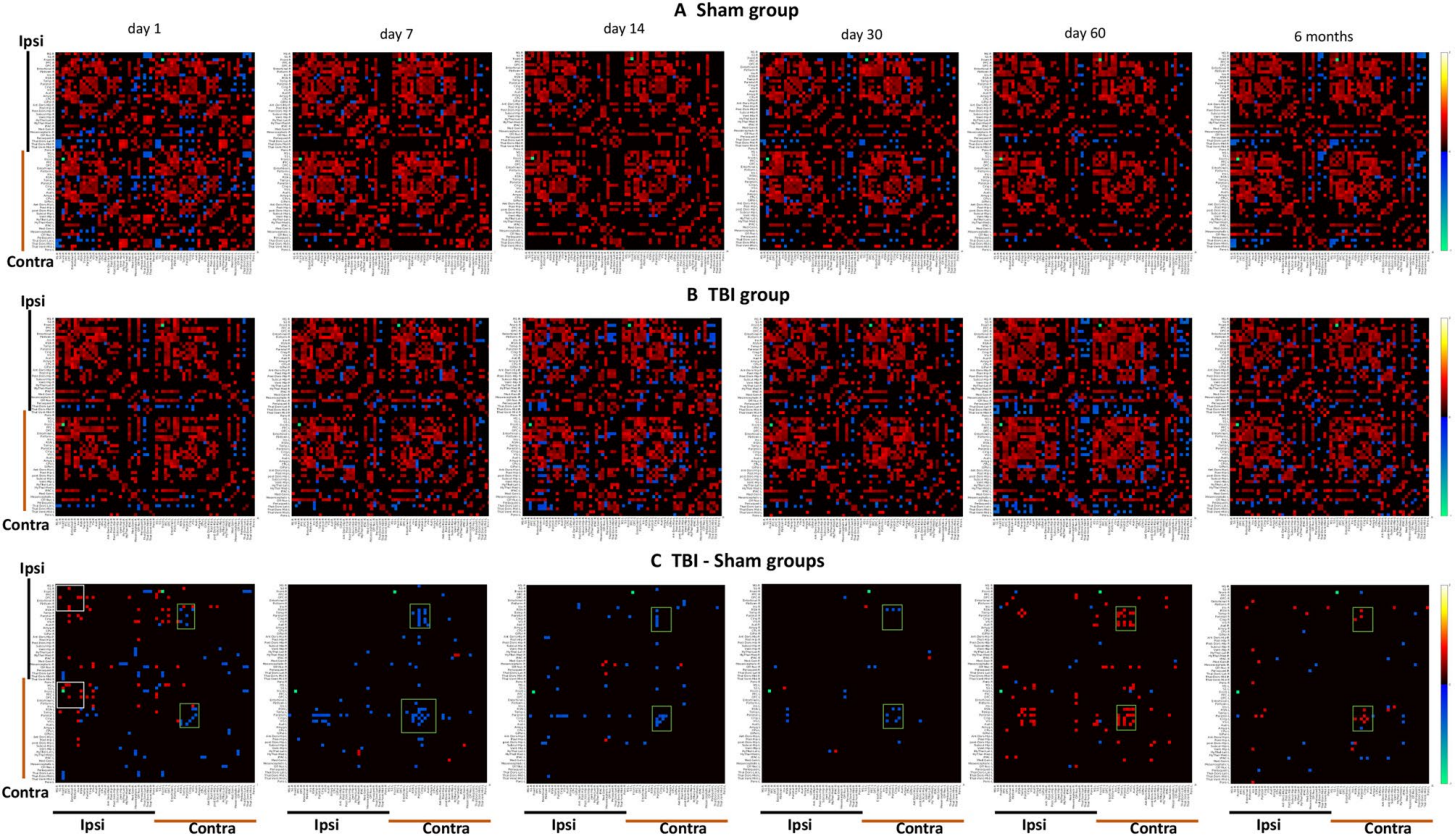
The functional connectivity analysis results revealed altered functional connectivity post-TBI. The mean functional connectivity matrices are presented as the one-sample t-test of correlation coefficients among seed regions for each group as (**A**) sham group and (**B**) TBI group. (**C**) The functional connectivity differences between the TBI group and sham group (estimated using unpaired t-test) showed mixed increased connectivity of Ipsilateral-ipsilateral and ipsilateral-contralateral sensorimotor cortex along with reduced functional connectivity of ipsilateral-contralateral and contralateral-contralateral seeds at day1. By day7, mainly reduced functional connectivity ipsilateral-contralateral and contralateral-contralateral seeds mainly within the default mode network (DMN) seeds, that tends to have a lesser extent by day30. At day60 the functional connectivity between the contralateral and ipsilateral was shown to increase and persist to six months, and most of the observed changes were at the cortical regions and the DMN seeds. The green boxes point to the DMN seeds, and the white boxes point to the sensorimotor networks. All results were corrected with false discovery rate (p ≤ 0.05).

Resting state functional connectome analysis produced a set of matrices representing the correlations of the time courses of different predefined seeds at different timepoints are presented in Fig. 4, including the mean connectivity of the sham group, TBI group, and differences between TBI and sham (FDR corrected, p ≤ 0.05). The results revealed significant connectivity differences in the TBI group compared to the sham group at day1, with relatively increased functional connectivity observed at day1 in the ipsilateral-ipsilateral and ipsilateral-contralateral sensorimotor regions and executive network seeds (FDR corrected, p ≤ 0.05). However, the ipsilateral-contralateral connectivity for default model network (DMN) seeds including cingulate cortex, temporal cortex and the retrosplenial cortex were decreased at day1 (p ≤ 0.05), and with the lowest connectivity of the ipsilateral-contralateral and contralateral-contralateral DMN observed at day7. This reduced connectivity tended to pseudo-recover slightly over time by day14 and up to day30 (p ≤ 0.05). By day60, the functional connectivity at ipsilateral-contralateral and contralateral-contralateral DMN connectivity were increased to its zenith (p < 0.01), which declined slightly at six months post-TBI (p ≤ 0.05).

## Discussion

In this work, we documented the time course of structural and functional alterations in the rat brain up to six months following a TBI as compared to sham injury animals. By utilising a multi-MRI approach (including morphometry, DTI, and resting state functional connectivity), we highlight a central position of grey matter regions corresponding to the DMN in this rather complex and dynamic process, which was associated with deficits in the integrity of critical white matter tracts, mainly the cingulum and corpus collosum. Indeed, DTI revealed the debut of reduced FA in the major white matter tracts at day1, which had persisted to six months after TBI (Table 1). In concert with reduced FA, we observed acute and subacute reductions in AD, and subacute and chronic increases in RD. These results indicate dynamic changes in white matter propagating with time after the injury (see Table 1), with distinct patterns at acute and chronic stages. This dynamic pattern likely reflects the ongoing pathologies, including the acute axonal injury and neuroinflammation, along with chronic neuroinflammation and demyelination.

**Table 1:**
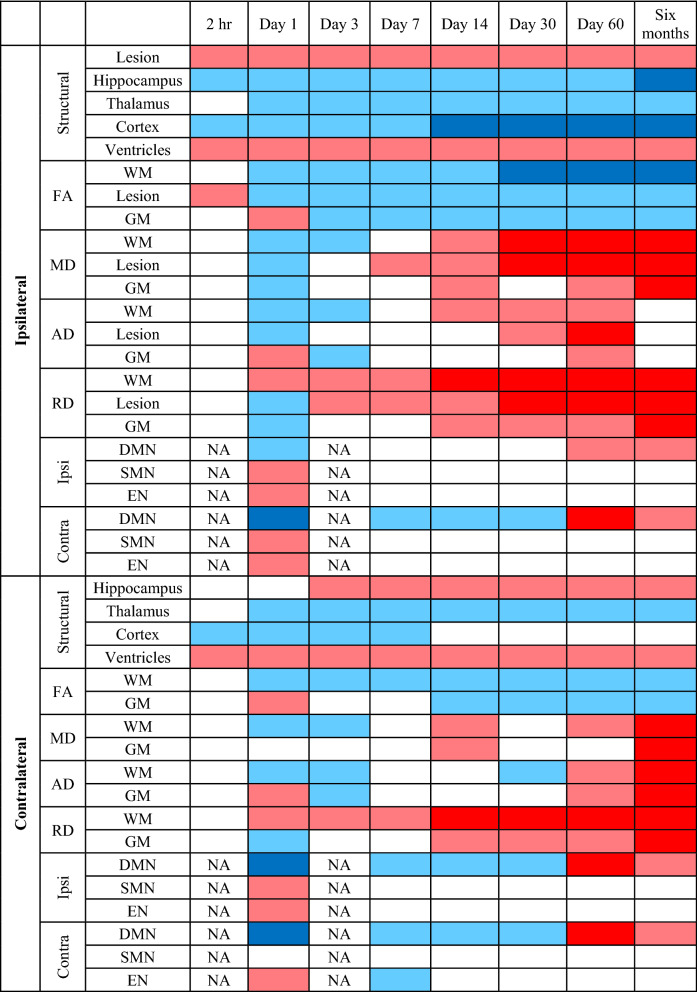
Results summary.

Ipsi, Ipsilateral; contra, Contralateral; FA, Fractional Anisotropy; MD, Mean diffusivity; AD, Axial diffusivity; RD, Radial diffusivity, CC, corpus callosum; NA, Not applicable, EN, Executive Network; SMN, Sensorimotor network; DMN, Default mode network; GM, grey matter; WM, white matter.

Colour codes:

Dark Blue: extreme reduction.

Light Blue: mild reduction.

Blank (no colour): No significant difference.

Pink: mild increase.

Red: extreme increase.

NA: Not applicable.

### Brain volume changes following CCI injury

The CCI model provoked a consistent injury profile, with T2-weighted hyperintensities appearing within the injury lesion in the first hours post-TBI, which diminished in the following days, suggesting the occurrence of acute and transient oedema^14,15^. Starting from day7, the lesion expanded spatially, eventually reaching the lateral ventricles at six months post-TBI, which is consistent with previous studies^14,15,34^. The progressive ventricular dilation has also been reported previously in several TBI animal models^26,35^, and in human TBI survivors^36–39^. We also observed severe hippocampal atrophy in the ipsilateral hemisphere, which we attribute to the direct and deep penetration properties of the CCI injury^21,22^. The hippocampus was also suggested to be vulnerable in the human TBI survivors^17–20^, who often suffer from persistent memory deficits^19,20^. Indeed, volume loss of the hippocampus, amygdala, and thalamus are strong predictors for functional impairments and disability post-TBI^40,41^. Such volume loss may result from an ongoing neuroinflammation following TBI^10,42^, which may impair hippocampal neurogenesis in rodents^10,42^ and human^43,44^.

### Altered microstructure following CCI injury in white matter tracts suggest ongoing axonal injury

Our results showed persistent reduction in FA in major white matter tracts are in accord with earlier rat findings up to 60 days post CCI^2,11,25^, and with human investigations within a few years post-TBI^23,24,45–48^. Present observations of reduced FA in the major white matter tracts persisting up to six months post-TBI were mainly driven by the acute reduction in AD (i.e., up to day3), and then by the subacute-chronic RD increases seen from day7 to six months (Table 1).

Acute reductions in FA and AD after TBI might reflect the transient aspects of axonal injury, such as axonal swelling^6,7^, along with the neuroinflammatory response occurring within hours to days post-injury^1,2,10^. The sub-acute and chronic reduction of FA and increased RD (i.e. from day7 onwards) may indicate a phase of demyelination followed by secondary microglial activation^2,49^. In rodents, the neuroinflammatory response was reported to peak at one week post-TBI^10^, while activated hypertrophic microglia were present at five, 12, and 52 weeks post-TBI^9,10^, and may persist up to one year^9,10,50,51^. In addition, the increased RD may reflect the ongoing demyelination process^52^, which has been previously thought to initiate at 7 days and persist for 28 days^2^, two months^11^, six months^12^, and even up to a year in rodent models post-TBI^13^. Thus, the present findings of increased RD seem to follow the same temporal profile shown in previous reports of demyelination post-TBI. In addition, the present findings of reduced FA and increased AD, MD, and RD at 6 months resemble those observed in male Vietnam War veterans with moderate-to-severe TBI who showed increased diffusivity measures and reduced FA when they were scanned decades post-trauma^53^. This concordance supports the translatability of the present chronic rodent study, in showing analogous findings of reduced volume of several cortical and subcortical regions, along with reduced FA and increased diffusivity measures persisting to six months post TBI.

From day14 onwards post-TBI, the increased diffusivity measure in the white matter may have resulted from the cortical neuron loss, whereby neurite density leads to axonal degeneration^1^. Indeed, we detected tissue loss that we initially confined to the lesion area, but proceeded to expand over time, presumably as a function of delayed neuronal loss. The lesion area also showed reduced diffusivity (including AD, RD, MD) at day1 post-TBI, which normalised at day3, but then increased from day7 onwards. The U-shaped temporal response in our model may reflect the acute cytotoxicity at day1, superseded by vasogenic oedema at day7 post-TBI, as reflected by high contrast on the T2-images and increased diffusivity at the lesion area, as also reported elsewhere^54^.

As for the rest of the grey matter —including hippocampus, thalamus, and cortex—the acute increase of FA and AD along with reduced RD at day1 post-TBI may reflect a phase dominated by cytotoxic oedema^55,56^. However, the reduced FA, driven by the increased RD and prevailing in grey matter from day3 and up to six months, might also reflect vasogenic oedema^54^, along with other net effects of neural loss, axonal injury, and removal of cellular debris as part of the neuroinflammatory response. Thus, we cannot be certain that the same factors account for the observed DTI alterations in both white matter and grey matter.

### Resting state functional connectivity networks following CCI

The temporal pattern of the microstructural changes in the brain described above occurred along with a significant time-dependent rearrangement of the functional connectivity between major networks, predominantly the DMN and the sensorimotor network. Previous animal studies showed similar functional connectivity alterations after CCI^27–30^ and in fluid-percussion brain injury^31,32^, which were suggested to result from the ongoing widespread microstructural alterations following the injury^31,32^.

In the acute phase of the injury, at day1, there was a clear increase in connectivity in the ipsilateral-ipsilateral primary motor and sensory cortices, and likewise in the perilesional cortical area. These findings are similar to those observed in Harris et al., who showed increased connectivity at day1 post-TBI followed by declining functional connectivity up to day28^27^. In addition, we observed declining functional connectivity in the DMN to a nadir at day7, followed by a pseudo-normalization by day30. The functional connectivity of the ipsilateral-contralateral and the contralateral-contralateral tended to increase to the peak by day60 and then slightly reduce by six months. This phasic response may track the neuroinflammatory response, which reportedly peaks at one week post-TBI^10^. Indeed, a previous human study showed a link between the disrupted functional connectivity (also mainly the DMN network) with ongoing neuroinflammation and worse cognitive deficits in TBI patients^57^. In a rodent fluid-percussion injury study, Mishra and colleagues reported decreased connectivity between ipsilateral and contralateral cortical regions at day7 post-TBI, with more pronounced effects on ipsilateral connectivity, occurring in conjunction with tissue loss^31^. Interruptions of the ipsilateral-contralateral functional connectivity as likewise seen in the current study, might arise from damage to the corpus callosum, as occurs following CCI^27–30,58^, as attested by decreased cerebral perfusion following CCI-TBI^59^.

The increased DMN connectivity at day60 and to a lesser extent at six months after TBI is supported by early findings in the DMN functional connectivity some weeks/months following injury^60,61^, which correlated with improving cognitive performance. Previous studies of functional connectivity in the rodent brain did not extend beyond 30 days post-injury^59^. Moreover, the human findings of recovered functional connectivity were mainly observed for ipsilateral-contralateral and contralateral-contralateral connectivity, which may suggest an ongoing neuroplasticity mechanism whereby homologous areas in the contralateral hemisphere contribute to or take over functions of the damaged side of the brain^62^, as similarly shown in stroke patients^63,64^. Delayed recovery of DMN connectivity is reported in human TBI survivors, and was suggested to indicate the onset of compensatory mechanisms contributing to the recovery of cognitive and attentional function post-TBI^65,66^. The present findings of increased connectivity at day60 and six months are coincident with the increased DTI diffusivity, which peaks on the contralateral side at these same time points. Nonetheless, the improvement in DMN connectivity was less pronounced at six months, possibly reflecting a pseudo-normalization of the DMN connectivity.

In Table 1, we have summarized the dynamics of the connectivity and volumetric changes in the form of a colour-coded depiction of different regions. The results showed very slight changes at two hours post-TBI, mainly proximal to the lesion. At the sub-acute stage, the changes suggest a cytotoxic oedema that transitions into vasogenic oedema along with other pathologies likely including neuroinflammation and demyelination. Most of these changes initiated ipsilateral to the lesion and then propagated to the contralateral hemisphere over time. This progression is supported by other studies CCI^2,11,25,27–30^. In addition, as shown in Table 1, most of the changes established by day14 persisted up to 6 months, except for the functional connectivity of the contralateral DMN, which was increased at day60 and likewise at 6 months. The functional connectivity increased at day1, decreased to a nadir at day7, and tended to normalise by day30, followed by a gradual recovery of contralateral connectivity up to 6 months. This profile of the altered functional connectivity follows a similar pattern of the observed alterations in the DTI, which showed a transient change peaking at day7. The dynamics could indicate an ongoing neuroplasticity of the injury brain in compensation for the various pathologies following TBI, whereby the gradual increase of connectivity could reflect a recovery of brain function.

We note several limitations of the present longitudinal and multimodal imaging investigation. First, we used male rats to create our model, since men are approximately 40% more likely to suffer a TBI as compared to women^67,68^, with women accounting for only 30% of TBI cases in Australia^69^. However, this may call for future investigations to identify possible gender effects on the brain structural and functional alterations following TBI^70^. Due to the longitudinal nature of our study, we performed neither histological nor immunohistochemical examinations, which limits our ability to draw inferences from the imaging findings about underlying biological mechanisms. However, there is already an extensive literature on the histopathological findings in the rat CCI model^3,71–74^. The lack of behavioural assessments makes it impossible to relate the ongoing alterations of the functional connectivity with behavioural recovery. Finally, the CCI model of TBI may not emulate the whole spectrum of human TBI pathology, for example with respect to the widespread axonal damage that occurs in human closed-head injury in the human.

## Conclusion

This is the first longitudinal MR imaging study extending to six months post injury to document the temporo-spatial pattern for changes in structural–functional integrity of the rat brain after TBI. These results confirmed the progression and persistence pathological cascades as reflected by the DTI alterations, and emphasises dynamic changes and recovery of the DMN. The results depict the net results of acute neurotoxic oedema and neuroinflammation responses and axonal injury followed by subacute and chronic processors of neuroinflammation, demyelination process, and neuronal loss. These results might suggest an ongoing neuroplasticity of the brain to compensate for the ongoing pathologies following TBI, since the changes in the functional connectivity tends to follow a similar trend to the observed changes in the DTI, with the increase of the functional connectivity to be a reflective of the brain performance to compensate of the ongoing pathologies.

## Methods

### Experimental design

**Figure 5:**
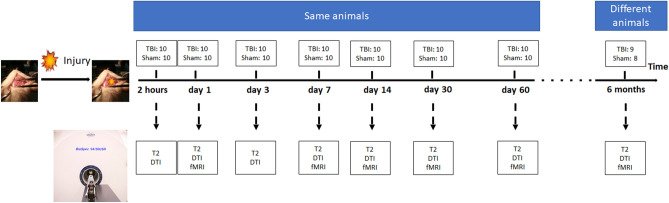
The experimental paradigm representing the experimental setup of the controlled cortical impact (CCI) project. Animals underwent the MRI imaging using T2 structural data, functional MRI, and diffusion tensor imaging (DTI) at two hours, days 1, 3, 7, 14, 30, and 60, and six months after the injury.

This study was approved by the Animal Research Ethics Committee (AEC) of the University of Queensland (IRB number: QBI/036/16/MAIC). Thirty-seven male Sprague–Dawley rats (8 − 10 weeks old, 300 − 340 g) were purchased from the Animal Resource Centre (ARC, Western Australia). Animals were housed two animals per cage, under conventional laboratory conditions with a 12-h light–dark cycle and free access to food and water. The animal quarters were maintained at 20 °C, and cages were cleaned twice a week. In the first phase, 20 rats were randomly assigned to receive sham surgery (n = 10) or CCI-TBI (n = 10), with sequential MRI scanning at 2 h, and at days 1, 3, 7, 14, 30, and 60 post-TBI (Fig. 5)s. A separate group of 17 rats was randomly assigned to sham (n = 8) and CCI (n = 9) groups for scanning at six months post-injury. All procedures in the current study were performed in accordance with the Animal Research- Reporting In Vivo Experiment guidelines (ARRIVE guidelines; https://arriveguidelines.org/), and the American Veterinary Medical Association (AVMA) Guidelines (https://www.avma.org/resources-tools/avma-policies/avma-guidelines-euthanasia-animals).

### Controlled cortical impact (CCI) injury model

For inducing the CCI model, rats were first anesthetised with isoflurane (5% for induction, 1–2% for maintenance) in 40% oxygen and placed in a stereotaxic frame. After incision of the scalp, a 5 mm diameter craniotomy was performed over the right hemisphere (centred at 3 mm posterior to the Bregma and 3 mm left lateral to the midline), without damaging the underlying dura. The CCI injury was applied in the TBI animals using an impactor (TBI 0310; Precision Systems and Instrumentation, USA) with a cylindrical piston tip 4 mm in diameter, operating at a velocity of 5 m/sec, and penetrating to a depth of 2 mm with a dwell time of 200 ms. After injury, the bone plug from the craniotomy was replaced and the scalp was sutured. Sham rats underwent the same procedure, but with omission of the impact. Rats showed no conspicuous signs of motor disability during monitored recovery.

### Magnetic resonance imaging procedure

Rats were anesthetised with isoflurane (4% induction, 1–2% during preparation, and 0–0.3% during scanning), and an infusion line was placed in the peritoneal cavity for the delivery of dexmedetomidine (Domitor, Pfizer, Karlsruhe, Germany) at the rate of 0.1 mg/kg/hr following an initial bolus dose of 0.1 mg/kg. During the MRI scan, animals’ body temperature was kept at 36 ± 1 °C using a thermostatically controlled heating blanket, and the respiration rate was in the range 60–95 breaths/minute. To accelerate recovery from dexmedetomidine after the MRI scan, animals received an intraperitoneal bolus dose of the α_2_-antagonist tipamezol (Antisedan, 0.1 mg/kg, Pfizer, Karlsruhe, Germany).

MRI scans were acquired with a 9.4 T Bruker system (BioSpec 94/30USR, Bruker, Germany) and ParaVision software (PV6.0.1, Bruker, Germany) using a four-element array coil, with approximately two hours per scan. Anatomical T2-weighted images were acquired using Rapid-Relaxation-with-Enhancement (RARE) with TR/TE = 5900/65 ms, RARE factors = 8, two averages, FOV = 32 × 25 mm, slice thickness = 0.5 mm, matrix size = 256 × 256 × 40. DTI images were collected using an axial echo-planar imaging (EPI) sequence with TR/TE = 10,000 / 29 ms, FOV = 24.8 × 24.8 mm, matrix size = 108 × 108 × 41, slice thickness = 0.5 mm, slice gap = 0.1 mm, 32 directions with b-values = 750, 1500 s/mm^2^, and four b_0_ volumes. The fMRI data were acquired using a standard, single shot, EPI sequence with TR/TE of 1000/15 ms, 10 dummy scans, matrix size = 64 × 64, FOV = 25.6 × 25.6 × 20 mm^3^, slice thickness = 1 mm, and with a total of 600 volumes of rsfMRI lasting for ten minutes. The fMRI scan was not acquired at the two hour and day3 time points.

### Data pre-processing

Data pre-processing and analysis were performed using the FMRIB Software Library (FSL 5.0.9; https://fsl.fmrib.ox.ac.uk/fsl/fslwiki), and advanced normalisation tools (ANTs) (Version: 2.1.0-gGIT-N).

T2-weighted images were skull stripped using the 3D pulse-coupled neural networks (PCNN)^75^, and were corrected manually. Next, the skull-stripped T2-weighted images were corrected for field bias inhomogeneity using ANTs-N4BiasFieldCorrection^76^ and spatially normalised to the Schwarz rat brain template^77^ using ANTS registration. The log Jacobian maps were generated for each animal at each scanning session using ANTS—*CreateJacobianDeterminantImage,* which was used for the tensor-based morphometry analysis.

The DTI data were corrected for eddy current (FSL-eddy correct) using the mean of the four b_0_ volumes as reference. To obtain the brain mask, DTI data were linearly registered to the T2 images (FSL*-flirt*), and the inverse registration matrix was applied on the T2-based mask to move the mask into the individual DTI space. The DTI images were corrected for field bias inhomogeneity (ANTs-N4BiasFieldCorrection). The FSL Diffusion Toolkit was used for local fitting of the diffusion tensors in a multi-shell approach to generate the different DTI measures maps, i.e., FA, RD, AD, and MD^78^. To allow for voxel-based group comparisons, the DTI maps were then normalised to the brain template by applying the field-deformation maps generated by the T2-to-template normalisation to the DTI maps.

The fMRI scans were corrected for slice-timing (FSL*-slicetimer*), spike removal (*3dDespike*), and head motion (FSL*-mcflirt*). The fMRI data were then linearly co-registered to the corresponding T2 images in the native space (FSL-*flirt*), which was used to register the brain mask for skull stripping. Motion artefacts and signals extracted from the white matter and ventricles were regressed out of the image files. The fMRI data were then spatially smoothed using a Gaussian kernel of 0.4 mm full width at half maximum (FWHM) to reduce spatial noise, and band-pass filtered (0.007 − 0.3 Hz). Finally, the fMRI data were normalised to the Schwarz brain template using the field-deformation maps generated from the T2-data normalisation to the template.

### Statistical analysis

To identify the temporo-spatial profile of differences in brain volume between the TBI and the sham groups over six months, we performed a voxel-based tensor-based morphometry analysis using the log-Jacobian maps as an input for voxel-wise analysis of variance (ANOVA) followed by a post hoc unpaired t-test. Similarly, to identify the temporo-spatial profile of the microstructural alterations over the six months post-TBI, we calculated differences between the TBI and sham animals in each of the DTI measures using voxel-based ANOVA, followed by an unpaired t-test at the different time points. This voxel-based analysis was performed using a permutation test with FSL-randomise^79^ and 5000 permutations. The results were corrected for multiple comparisons using Family Wise Error (FWE; p ≤ 0.05, cluster size > 50 voxels).

In addition to structural and microstructural alterations, the seed-to-seed functional connectivity analysis of the fMRI data was used to generate the functional connectome matrix for each animal at each MRI session, and then used to compute a one-sample group average of each group and unpaired t-test contrasting TBI vs. Sham groups at different time points. This functional connectome analysis was performed using FSLNETs, which calculated the Pearson's correlation coefficients between the mean time-courses from each region of interest (ROI), as per the segmentation of the Schwarz template. The Pearson's correlation coefficients were then translated to z-values by Fisher’s z-transform, and the group mean and difference between groups was determined by again using the permutation test (FSL-randomise) with 5000 permutations. Significance was determined using the false discovery rate (FDR) with p ≤ 0.05.

The regions of interest (ROIs) used to generate the functional connectome consisted of 33 bilateral brain regions. These regions were motor (M1), somatosensory (S1), frontal, medial prefrontal (PFC), orbito-frontal (OFC), entorhinal, piriform, insular (Ins), retrosplenial (RSN), temporal (Temp), parietal, cingulate (Cing), visual, and auditory cortex, as well as, amygdala (Amyg), caudate-putamen (CPu), Globus-Pallidus (GlPal), antero-dorsal hippocampus (Ant-Dors-Hip), posterior hippocampus (Post-Hip), postero-dorsal hippocampus (Post-Dors-Hip), subiculum hippocampus (Subcul-Hip), ventral hippocampus (Vent-Hip), lateral hypothalamus (HyThal-Lat), medial hypothalamus (HyThal-Med), anterior commissure (IPAC), medial geniculate (Med-Gen), mesencephalic region (Mesencephalic), olfactory nuclei (Olf-Nuc), periaqueductal grey (Periaqued), dorsolateral thalamus (Thal-Dors-Lat), dorsomedial thalamus (Thal-Dors-Mid), and ventromedial thalamus (Thal-Vent-Mid), and pons.
